# Propofol vs. inhalational agents to maintain general anaesthesia in ambulatory and in-patient surgery: a systematic review and meta-analysis

**DOI:** 10.1186/s12871-018-0632-3

**Published:** 2018-11-08

**Authors:** Stefan Schraag, Lorenzo Pradelli, Abdul Jabbar Omar Alsaleh, Marco Bellone, Gianni Ghetti, Tje Lin Chung, Martin Westphal, Sebastian Rehberg

**Affiliations:** 1 Department of Perioperative Medicine, Golden Jubilee National Hospital, Clydebank, G81 4DY Scotland, UK; 2 AdRes-Health Economics and Outcome Research, Torino, Italy; 3 Fresenius Kabi Deutschland GmbH, Bad Homburg, Germany; 4 Department of Anaesthesiology, Intensive Care and Pain Medicine, University Hospital, Münster, Germany; 5 Department of Anaesthesiology, University Hospital, Greifswald, Germany; 6 Department of Anaesthesiology, Intensive Care, Emergency, Transfusion and Pain Medicine, Evangelisches Klinikum Bethel, Bielefeld, Germany

**Keywords:** Propofol, Sevoflurane, Desflurane, Isoflurane, Total Intravenous Anaesthesia, Inhalational Anaesthesia, Postoperative Nausea and Vomiting, Postoperative Pain, Target Controlled Infusion, Patient Reported Outcomes

## Abstract

**Background:**

It is unclear if anaesthesia maintenance with propofol is advantageous or beneficial over inhalational agents. This study is intended to compare the effects of propofol vs. inhalational agents in maintaining general anaesthesia on patient-relevant outcomes and patient satisfaction.

**Methods:**

Studies were identified by electronic database searches in PubMed™, EMBASE™ and the Cochrane™ library between 01/01/1985 and 01/08/2016. Randomized controlled trials (RCTs) of peer-reviewed journals were studied. Of 6688 studies identified, 229 RCTs were included with a total of 20,991 patients. Quality control, assessment of risk of bias, meta-bias, meta-regression and certainty in evidence were performed according to Cochrane. Common estimates were derived from fixed or random-effects models depending on the presence of heterogeneity. Post-operative nausea and vomiting (PONV) was the primary outcome. Post-operative pain, emergence agitation, time to recovery, hospital length of stay, post-anaesthetic shivering and haemodynamic instability were considered key secondary outcomes.

**Results:**

The risk for PONV was lower with propofol than with inhalational agents (relative risk (RR) 0.61 [0.53, 0.69], *p* < 0.00001). Additionally, pain score after extubation and time in the post-operative anaesthesia care unit (PACU) were reduced with propofol (mean difference (MD) − 0.51 [− 0.81, − 0.20], *p* = 0.001; MD − 2.91 min [− 5.47, − 0.35], *p* = 0.03). In turn, time to respiratory recovery and tracheal extubation were longer with propofol than with inhalational agents (MD 0.82 min [0.20, 1.45], *p* = 0.01; MD 0.70 min [0.03, 1.38], *p* = 0.04, respectively). Notably, patient satisfaction, as reported by the number of satisfied patients and scores, was higher with propofol (RR 1.06 [1.01, 1.10], *p* = 0.02; MD 0.13 [0.00, 0.26], *p* = 0.05). Secondary analyses supported the primary results.

**Conclusions:**

Based on the present meta-analysis there are several advantages of anaesthesia maintenance with propofol over inhalational agents. While these benefits result in an increased patient satisfaction, the clinical and economic relevance of these findings still need to be addressed in adequately powered prospective clinical trials.

**Electronic supplementary material:**

The online version of this article (10.1186/s12871-018-0632-3) contains supplementary material, which is available to authorized users.

## Background

It is estimated that of the more than 300 million surgical procedures undertaken worldwide annually, the majority requires anaesthesia services [1]. Propofol and volatile anaesthetic agents are the key components of modern general anaesthesia and account for many beneficial outcomes in clinical anaesthetic practice and in perioperative medicine [2]. Previous attempts have been made by systematic reviews and meta-analyses to establish particular benefits for one technique over the other with mixed or inconclusive results, but concerns were also raised which methodology is able to best capture these [3]. It is still not known in many domains whether or not propofol-based total intravenous anaesthesia (TIVA) techniques are more advantageous to patients than volatile-based anaesthesia. Often, surrogate and composite endpoints have precluded clinical application of results [4]. In addition, patient-reported outcome measures (PROM) are increasingly relevant to health care providers and will influence clinical choices [5]. We have therefore undertaken a new exhaustive and comprehensive literature review representing a wide spectrum of clinical applications on key parameters relevant to patient outcome, in particular the influence of choice of anaesthetic technique on post-operative nausea and vomiting (PONV) [6]. Potential confounders and bias were minimised by strict adherence to Cochrane guidelines [7].

We conducted a systematic review and meta-analyses of randomized controlled trials (RCTs) on the efficacy and safety of propofol (TIVA) vs. inhalational agents in maintaining general anaesthesia in ambulatory and in-patient surgical procedures. We also studied how those results vary by type of comparator, patient characteristics, type and setting of surgery. A systematic literature review of RCTs published up to date of the search was performed and predefined outcomes from selected studies were extracted, pooled and meta-analysed. A secondary meta-analysis also included observational studies. Reporting was done according to the PRISMA reporting guideline [8].

This systematic review was registered at PROSPERO, 22 July 2016, registration number CRD42016039539.

## Methods

The systematic literature review and meta-analyses were performed and reported according to the Cochrane Handbook [7] and Preferred Reporting Items for Systematic Reviews and Meta-Analyses (PRISMA-P, PRISMA) guidelines [8, 9]. The quality of evidence for all outcomes was judged using the Grading of Recommendations Assessment, Development and Evaluation working group methodology (GRADE) [10]. A full and detailed description of the methodology and the search strings can be found in an additional file (Additional file 1).

### Study identification and selection

We performed a literature search to identify published studies investigating the efficacy and safety of propofol vs. inhalational anaesthetics as single maintenance agents for general anaesthesia in the setting of in/out-patient surgery. Scientific articles in English, Italian, German, Spanish, and French reporting original data of RCTs (primary analysis) and observational studies (secondary analysis) published in peer-reviewed journals were evaluated. Studies conducted on adult and paediatric patients were included that used sevoflurane, desflurane or isoflurane as volatile agents. We excluded studies where study design and methodology were unclear or did not sufficiently describe the intervention, on non-target populations (e.g. ICU sedation), or if a non-standardised delivery protocol was used. Duplicates and publications reporting non-reliable data or for which only the abstract was available were excluded. We designed the search strategies according to the PICOS (Patients, Intervention, Comparator, Outcome, Settings) algorithm and employed medical subject headings (MeSH). The keywords for the search for RCTs were: “volatile gas”, “inhalation anaesthesia”, “sevoflurane”, “desflurane”, “isoflurane”, “propofol”, “randomized controlled trial”. There were no restrictions or filters used. We searched MEDLINE (Pubmed interface), EMBASE (Elsevier interface), and the Cochrane Central Register of Controlled Trials (Wiley interface). The coverage interval for the search was January 1st, 1985 to August 1st, 2016. A confirmatory search in PubMed and EMBASE was performed in November 2016 but yielded no further relevant results.

All identified publications were screened based on title and abstract. If studies were assessed as potentially eligible the full text was subsequently reviewed. Finally, the reference lists of included studies and previous reviews and meta-analyses on the research topic were manually checked to ensure literature saturation.

### Data extraction and quality assessment

Data with regard to study population, sample size, interventions, comparators, potential biases in the conduct of the trial, and outcomes were extracted from eligible publications.

The Cochrane Collaboration tool was used to assess the risk of bias (high/low) and covered the aspects: random sequence generation, allocation concealment, blinding of participants and personnel, blinding of assessments, attrition bias and selective reporting. If there was insufficient detail reported in the study, we judged the risk of bias as ‘unclear’. The assessment was performed independently by two reviewers based on The Cochrane Handbook 2011.

### Outcomes

For this review, the meta-analysis considered PONV as primary outcome. Secondary, patient-relevant outcomes were time to recovery (awakening/respiratory recovery/ orientation/reach a recovery score/follow simple instructions), post-operative pain, agitation on emergence, post-anaesthetic shivering, hospital length of stay and haemodynamic instability requiring interventions. Additional outcomes on efficacy, safety and resource consumption are listed in an additional file (Additional file 1).

### Statistical analysis

Quantitative synthesis was performed for data from eligible RCTs as primary meta-analysis. A secondary meta-analysis included information from additional observational studies. Common estimates were derived from fixed-effect models using the inverse-variance and Mantel-Haenszel approach for continuous and dichotomous outcomes, respectively [11]. In the presence of high heterogeneity, random-effects models were applied using the DerSimonian and Laird method [12]. The difference of means with 95% confidence intervals (95% CI) was calculated for continuous outcomes. In case of different measurement scales the standardized mean difference was used. For dichotomous outcomes, the risk ratio (RR) with 95% CIs was calculated. We considered *P* ≤ 0.05 to be statistically significant.

For RCTs, a comprehensive set of subgroup analyses were a-priori defined to identify differences among different volatile agents, patient characteristics, types of surgery, induction regimens, in/out-patients, and use of target-controlled infusion (TCI). The domains studied were selected by the authors when considered clinically relevant in relationship to the respective clinical outcome. These analyses were to be performed for subgroups that had a minimum of 10 studies.

We generated funnel plots in order to identify publication bias. The statistical analyses were performed using the software Review Manager (RevMan 5) and R (version 3.4.1, 2017-06-30, The R Foundation for Statistical Computing).

## Results

### Study selection

**Fig. 1:**
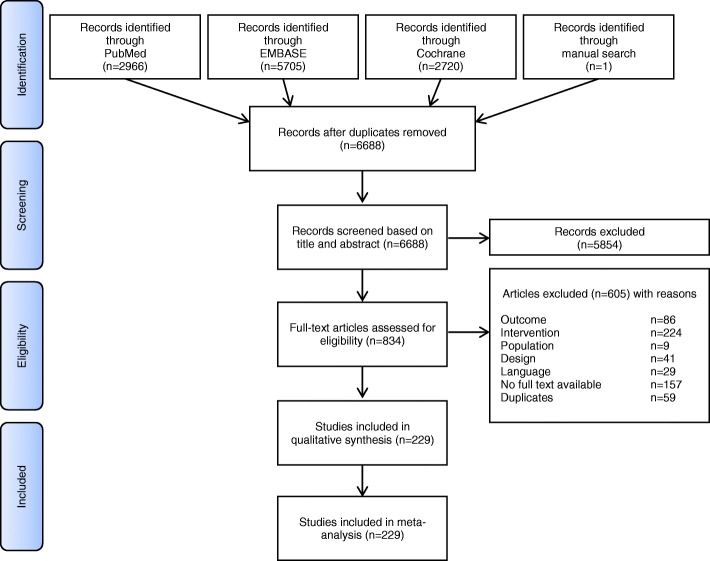
Study selection process of randomized controlled trials

The search of three databases resulted in 11,391 records, one further RCT was identified by checking manually the reference list of included papers. After exclusion of duplicates, 6688 records were screened based on title or abstract, whereof 5854 were excluded. After the detailed review of 834 full-text articles, we found that 86 did not report any outcome of interest, in 224 trials the intervention was not in accordance with the inclusion criteria, 9 did not fulfil the population criteria, 41 were not RCTs or had an unclear design, 29 reported in a non-selected language, for 157 no full text was available, and 59 were duplicates. Finally, 229 RCTs were included in the qualitative synthesis and primary meta-analysis (Fig. 1). These studies account for a total of 20,991 patients. An additional file (Additional file 2) describes the number of included RCTs with full descriptors by year of publication, inhalational agent, type of surgery and in/out-patient setting in more detail.

### Risk of bias

Overall analysis reveals a moderate risk of bias; however, it was unclear in the domains for randomisation, allocation and blinding. In particular, the most critical domains are those related to allocation concealment and blinding (Table 1). The primary outcome, however, was associated with a low risk of bias, as it was least influenced by blinding of outcome assessment and random sequence generation.

**Table 1:** Summary of risk of bias assessment of randomized controlled trials

Domain	Low risk	High risk	Unclear
Random sequence generation	46%	< 1%	53%
Allocation concealment	12%	30%	58%
Blinding of patients and personnel	17%	31%	53%
Blinding of outcome assessment	23%	23%	54%
Incomplete outcome data	96%	< 1%	4%
Selective reporting	95%	< 1%	4%

### Synthesis of results

**Fig. 2:**
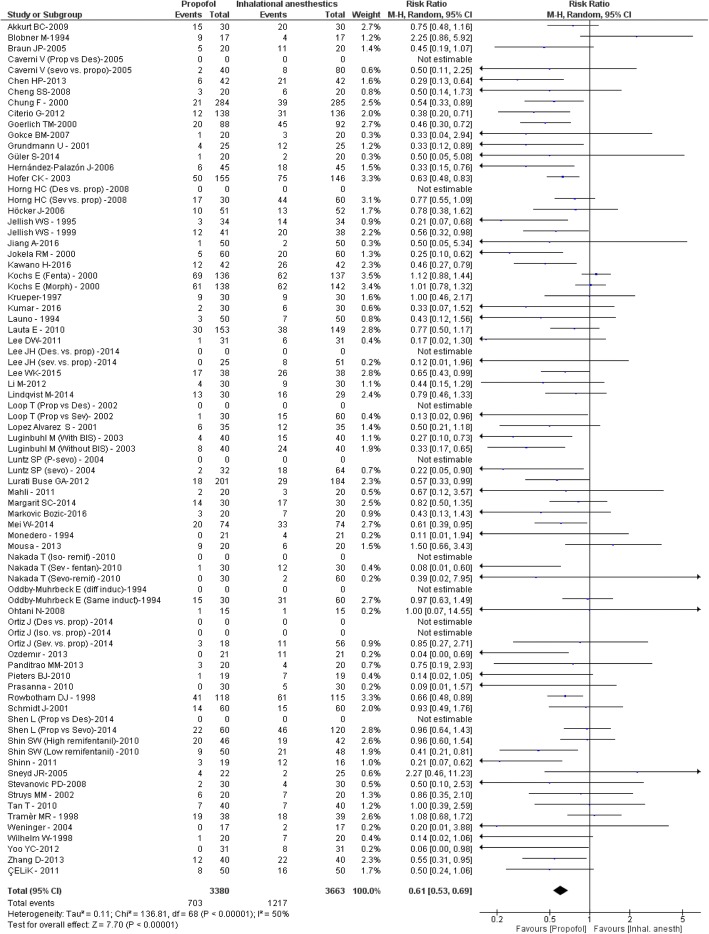
Forest plot of PONV for propofol and inhalational anaesthetics of the primary meta-analysis. Events refer to the number of patients with PONV. The risk ratio was used as effect size (PONV risk for the propofol group divided by PONV risk for the control group). Random effect models were applied to calculate a common estimate using the Mantel-Haenszel approach (Abbreviations: *M-H* Mantel-Haenszel approach, *CI* confidence interval)

For the primary outcome PONV, a statistically significant 39% reduction of the relative risk was observed for propofol as compared to volatile agents (effect estimate 0.61, 0.53–0.69 95%CI, *p* < 0.00001) as expressed in the respective forest plot (Fig. 2). This effect remained significant in all subgroups and was independent from the induction regimen as shown in an additional file (Additional file 3).

Among the secondary outcomes, mean differences of immediate recovery indicators as well as a lower consumption of muscle relaxants were significantly in favour of inhalational anaesthetics and associated with a faster respiratory recovery (0.82 min, 0.20–1.45 95%CI, *p* = 0.01) and time to tracheal extubation (0.70 min, 0.03–1.38 95%CI, *p* = 0.04). This, however, did not translate into shorter PACU times. On the contrary, the use of propofol shortened the mean time spent in PACU by − 2.91 min (− 5.47 to − 0.35 95%CI, *p* = 0.03).

Patients who received propofol for general anaesthesia experienced less pain post-operatively. Patient satisfaction, as a relevant patient-reported outcome measure, was significantly in favour of propofol as suggested by a higher number of satisfied patients as well as better satisfaction scores.

Data on hospital mortality including 11 studies revealed a common risk ratio of 0.78 (0.40–1.53 95%CI), which did not indicate a statistical difference between propofol and inhalational anaesthetics. A summary of the effect measures of all primary and secondary outcomes can be found in Table 2.

**Table 2:** Summary of effect estimates for primary and secondary outcomes in the primary meta-analysis

Outcome	No. of studies	No. of patients	Effect measure (statistical model)	Effect estimate (95% CI)	Effect
Haemodynamic instability	14	1327	RR (M-H, FE)	1.01 [0.86, 1.19]	NS
Accidental awakenings	3	350	RR (M-H, FE)	1.36 [0.64, 2.89]	NS
Muscle relaxant consumption	11	821	Std. MD (IV, FE)	0.18 [0.04, 0.32]	I
Agitation on emergence	6	923	RR (M-H, FE)	0.91 [0.61, 1.34]	NS
Time to awakening (min)	40	3064	MD (IV, RE)	−0.01 [− 0.71, 0.68]	NS
Time to respiratory recovery (min)	20	2622	MD (IV, RE)	0.82 [0.20, 1.45]	I
Time to orientation (min)	20	1738	MD (IV, RE)	1.35 [−0.12, 2.82]	NS
Time to reach a recovery score (min)	12	2114	MD (IV, RE)	0.13 [−1.09, 1.35]	NS
Time to follow simple instructions (min)	23	2497	MD (IV, RE)	0.46 [−0.39, 1.30]	NS
Time to tracheal extubation (min)	61	5773	MD (IV, RE)	0.70 [0.03, 1.38]	I
Post-anaesthetic shivering	23	3382	RR (M-H, FE)	1.06 [0.92, 1.22]	NS
PONV	65	7043	RR (M-H, RE)	0.61 [0.53, 0.69]	P
Pain score - After tracheal extubation	27	2631	MD (IV, RE)	−0.51 [−0.81, −0.20]	P
Pain score - 12 to 24 h after tracheal extubation	18	1836	MD (IV, RE)	−0.14 [− 0.31, 0.03]	NS
Analgesic consumption	40	4192	Std. MD (IV, RE)	0.20 [0.03, 0.37]	I
PACU time (min)	21	2653	MD (IV, RE)	−2.91 [−5.47, −0.35]	P
Cognitive assessment scales	7	387	Std. MD (IV, RE)	0.45 [−0.15, 1.05]	NS
Post-operative cognitive dysfunction	4	674	RR (M-H, FE)	1.14 [0.86, 1.51]	NS
Patients satisfied	10	924	RR (M-H, FE)	1.06 [1.01, 1.10]	P
Satisfaction score	5	967	Std. MD (IV, FE)	0.13 [0.00, 0.26]	P
Grade 3–4 adverse event	14	2046	RR (M-H, FE)	1.04 [0.76, 1.42]	NS
Hospital mortality	11	1427	RR (M-H, FE)	0.78 [0.40, 1.53]	NS
Hospital LOS (days)	30	2837	MD (IV, RE)	0.18 [−0.16, 0.53]	NS

*Abbreviations*: *PONV* post-operative nausea and vomiting, *PACU* post-anaesthesia care unit, *LOS* length of stay, *CI* confidence interval, *RR* relative risk, *MD* mean difference, *Std. MD* standardized mean difference, *M-H* Mantel-Haenzsel, *FE* fixed effects, *IV* inverse variance, *RE* random effects, *P* in favour of propofol, *I* in favour of inhalational agents, *NS* not significant

**Fig. 3:**
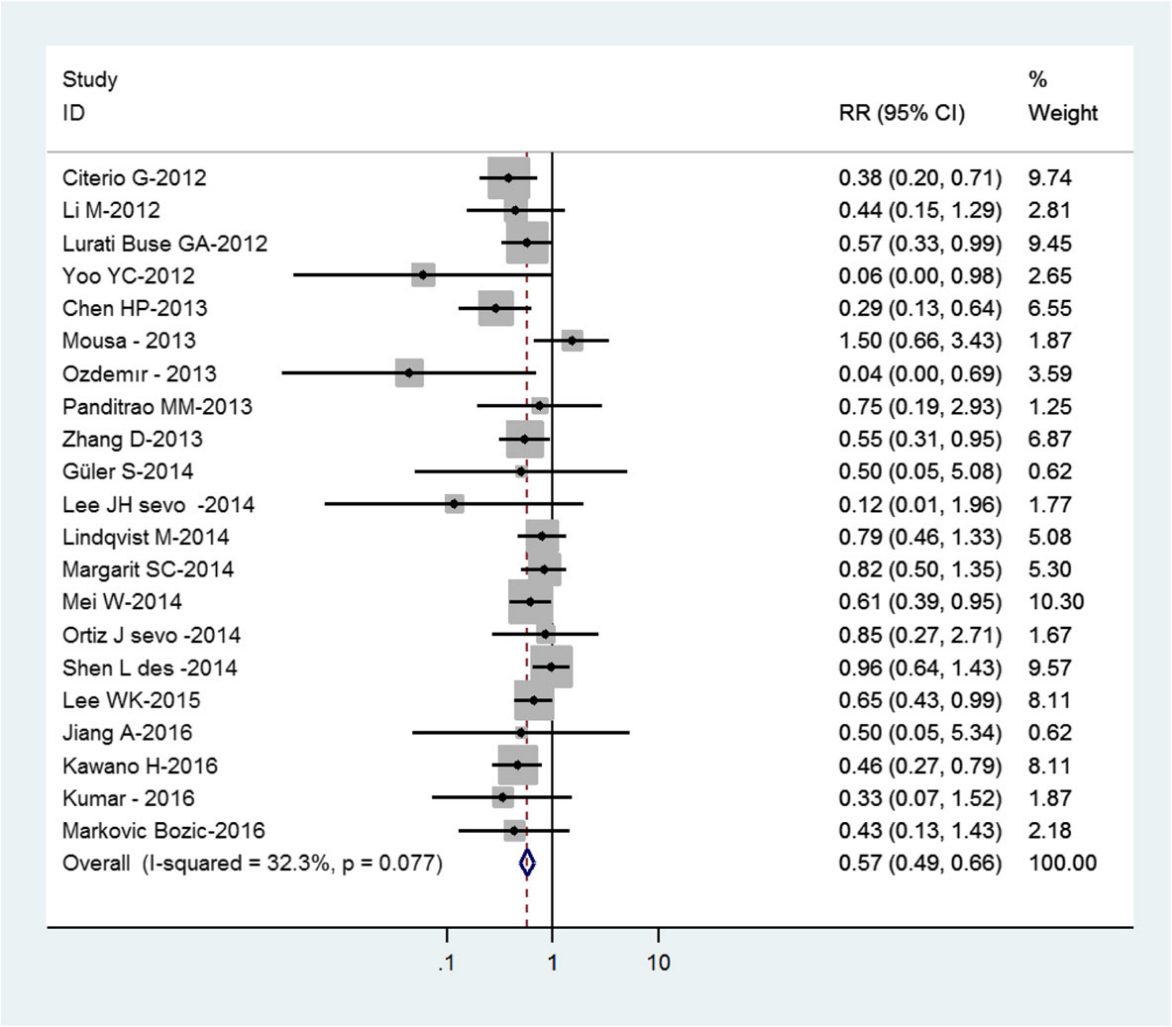
Summary of the sensitivity analysis on PONV. This Forest plot shows results of 21 studies between 2012 and 2016 (RR relative risk with 95% confidence interval)

In order to check whether or not incidence of PONV has changed in recent years, we have selected studies published between 2012 and 2016 on PONV. Of all RCTs, 21 studies fell into this period and were seperately meta-analyzed. The PONV incidence does not seem to have changed in the more recent studies as the raw incidence in control groups was 31% vs 33% in the overall analysis. Treatment effect was absolutely consistent with a relative risk of 0.57, exactly central to the original confidence interval. The Forest plot of this subgroup is shown in Fig. 3.

Advantages for propofol over volatiles could be demonstrated throughout subgroups for PONV. Evidence for lower pain scores could be seen for propofol use in the overall analysis, and in the subgroups of laparoscopic and in-patient surgical procedures.

We did not notice an influence of target-controlled infusion (TCI) for propofol as a contributing factor to faster recovery (i.e., time to awakening/respiratory recovery/tracheal extubation). Some differences in outcomes (time to orientation/follow simple instructions) were detectable only in subgroups, but not in the overall analysis (Additional file 3). There were no significant differences found in other subgroups.

A full report of the results and data disclosure can be requested from the authors.

## Discussion

The most important finding of this meta-analysis is a consistent and clinically relevant reduction on PONV when using TIVA over volatile maintenance of general anaesthesia. The relative risk reduction of 39% in our study is more than twice as high as the 18.9% reported by Apfel and colleagues in 2004 [13]. Although the anti-emetic effect of propofol is widely known [14] and documented elsewhere, the adoption of this as a first line technique to minimise PONV has not been routinely translated into clinical practice. In spite of grade A1 evidence to reduce baseline risk of PONV in recent consensus guidelines [15] the uptake of TIVA as a default anaesthetic technique is still slow but should be recommended based on our results. For instance, it is estimated that less than 20% of all general anaesthetics in the UK use propofol as maintenance [16]. The present results re-emphasize the range of advantages of propofol over inhalational anaesthetics, including the lower requirements of postoperative analgesia.

In contrast, most previous systematic reviews comparing TIVA with volatile anaesthetics were primarily focused on, and therefore limited to specific clinical domains, like ambulatory surgery [17], cardiac surgery [18, 19], neurosurgery [20], post-operative pain [21, 22], post-operative complications [23], robotic surgery [24] or were even looking at differences in survival [25]. In addition, some of the reviews significantly date back in time more than 20 years [26].

We found, as a secondary outcome, a moderate but consistent effect on lower pain scores by intravenous anaesthesia compared to volatile anaesthetics. Although associated with higher intraoperative use of analgesia, patients receiving TIVA experienced better pain control immediately after surgery (*p* = 0.001) and after 24 h (*p* = 0.1). Recently, some evidence has emerged linking intraoperative propofol exposure with an intrinsic analgesic effect [27] demonstrated as reduced post-operative analgesic requirements [28] and the absence of hyperalgesia [29]. However, in an attempt to address this in a systematic review and meta-analysis, Peng and colleagues recently found substantial heterogeneity among studies, limiting their conclusions [22].

Both PONV and post-operative pain are significant determinants of patient dissatisfaction as Myles and colleagues found in validating the quality of recovery score 40 (QoR40) [30]. Of all recovery domains analysed in a subsequent cohort study only pain and PONV contributed to incomplete patient satisfaction [31] whereas TIVA improves early patient well-being by reducing PONV and pain [32] dependent on the setting [33]. This hypothesis is supported by increased patient satisfaction with propofol in the present study. Another factor, influencing patient outcome is the incidence of post-operative agitation and delirium. In this context, it is noteworthy that after adding three observational studies (4387 patients) in the secondary analyses, there was a reduced risk for agitation with propofol. There are indicative studies associating volatile agents with the development neuro-cognitive dysfunction [34, 35], although an earlier meta-analysis could not demonstrate an association of general anaesthesia per se and risk of Alzheimer’s disease [36].

We found no difference between anaesthetic techniques for other outcomes such as, post-anaesthetic shivering, hospital length of stay or haemodynamic instability requiring unplanned interventions. Although time to tracheal extubation and respiratory recovery was significantly faster for volatile anaesthesia (on average one minute) and time in PACU significantly shorter for TIVA (on average three minutes), we would consider neither as clinically relevant. The lack of difference in these secondary outcomes might be explained in the varying standards and definitions of reporting the criteria in the individual studies as well as other procedure related factors that, independent of the anaesthetic technique, might have influenced outcomes. Some of the main and secondary outcomes were consistent in the domains of the subgroup analyses and in favour of propofol maintenance. Others, like differences in immediate recovery milestones, type of surgery, haemodynamic instability were not. There has been a debate in recent years whether or not volatile anaesthesia offers advantages in survival due to myocardial events in cardiac surgical [37–39] and non-cardiac patients [23, 40]. This systematic review did not demonstrate any contribution of the anaesthetic technique to perioperative survival, although we did not specifically include perioperative myocardial events or its impact on 1-year mortality. The malignancy status of patients undergoing oncological surgical procedures as a contributing factor for differences in outcome of observed parameters could also not be established by our data material although in a recent retrospective database trial an association of survival and type of anaesthetic could be demonstrated [41].

We have to consider some limitations to our analysis and report. Firstly, although large in number, the publications included in our systematic review and meta-analysis represent the scientific output across three decades. Clinical practice is continuously changing and adapting. Older publications may therefore no longer be representative of current scientific consensus and clinical practice. However, over 80% of the studies included data from 2001 onwards and 40% were published between 2011 and 2016 (Additional file 2). Nevertheless, the combination of varying conditions and settings and their heterogeneity into one estimate may limit the clinical interpretation when using a random effects model. Secondly, although the Cochrane tool for assessing risk of bias was systematically applied in the analysis, we have identified some areas of moderate risk of bias (Table 1) [42, 43] as well as domains with a low number of studies in the subgroup analysis. This needs to be considered when interpreting the results [44]. Based on the studies available, there is a lack of RCTs specifically comparing TCI with non-TCI techniques for propofol maintenance and its impact on meaningful outcome parameters. We could not establish enough study power for this comparison in the available collection of RCTs. A previous Cochrane review on TCI has recently been withdrawn owing to the age, retraction of studies, and possible errors in the conversion of dose for the purpose of analysis [http://cochranelibrary-wiley.com/doi/10.1002/14651858.CD006059.pub3/full].

## Conclusion

Patient-reported outcomes after surgery is becoming increasingly relevant for health care providers to improve quality of care and to define key performance indicators in perioperative medicine. Reviewing 229 RCTs with 20,991 patients from peer-reviewed publications including a wide variety of clinical specialties and patient populations, we found reduced PONV and better analgesia with TIVA as compared to general anaesthesia with volatile anaesthetics. Although patient discomfort after surgery is common, it is less well understood how it affects patient satisfaction [45]. This large systematic review and meta-analysis shows that in a relevant clinical domain of perioperative care TIVA provides a better patient experience compared to volatile anaesthesia with a high degree of confidence in the effect estimate [46]. These promising findings will hopefully trigger the conduct of prospective validation studies.

## Additional files

Additional file 1: This file explains the methodology of the meta-analysis and systematic review in more detail. (DOCX 48 kb)

Additional file 2: This file gives additional information on the characteristics of all studies included in the primary meta-analysis. (DOCX 134 kb)

Additional file 3: This file provides a summary of effect estimates for primary and secondary outcomes including subgroups of all RCTs. (DOCX 36 kb)

## Abbreviations

BIS: Bispectral index
CI: Confidence interval
FE: Fixed effect
GRADE: Grading of recommendations assessment, development and evaluation working group methodology
ICU: Intensive care unit
IV: Inverse variance
LOS: Length of stay
MD: Mean difference
MeSH: Medical subject headings
M-H: Mantel-Haenszel
N/R: Not reported
NNT: Number needed to treat
OS: Observational study
p: *p*-value
PACU: Post anesthesia care unit
PICOS: Patients, intervention, comparator, outcome, settings
PONV: Post-operative nausea and vomiting
PRIS: Propofol infusion syndrome
PRISMA: Preferred reporting items for systematic reviews and meta-analysis
PROM: Patient-reported outcome measure
RaR: Rate ratio
RCT: Randomized clinical trial
RE: Random effect
RN satisfaction score: Result of the Registered Nurse survey by the Nursing Quality Indicators® (NDNQI®)
RR: Risk ratio
SD: Standard deviation
SEM: Standard error of the mean
SGA: Subgroup analysis
SPC: Summary of product characteristics
St. MD: Standardized mean difference
TCI: Target controlled infusion
TIVA: Total intravenous anesthesia
