# Supplementary material for: Propofol vs. inhalational agents to maintain general anaesthesia in ambulatory and in-patient surgery: a systematic review and meta-analysis
Source: BMC Anesthesiol. 2018 Nov 8;18:162. doi: 10.1186/s12871-018-0632-3 (PMC6225663; doi:10.1186/s12871-018-0632-3)
Supplement: Supplementary file 1 — This file explains the methodology of the meta-analysis and systematic review in more detail. (DOCX 48 kb) [file 12871_2018_632_MOESM1_ESM.docx]

**Additional file 1**

**_________________________________________________________________________________**

M**ETHODS**

The methods to be followed were described in the protocol registered at PROSPERO (https://www.crd.york.ac.uk/prospero/), 22 July 2016, registration number CRD42016039539.

A structured literature search was performed to identify publications investigating the efficacy and safety of propofol vs. inhalational anaesthetics as single maintenance agents for general anaesthesia, in the setting of ambulatory or in-patient surgery. For studies meeting the defined criteria for inclusion, data on descriptive, effectiveness, and safety parameters were extracted in a structured data grid.

# Eligibility criteria

**Report characteristics**

Papers published in one of the following languages were evaluated: English, Italian, German, Spanish, French. Studies for which only the abstract was available, duplicates and non-reliable data (e.g., retracted papers) were excluded. The publication date had to be between 1 January 1985 (first report on a clinical comparison of propofol vs. inhalational anaesthetic) and the date of the last search (i.e., 1 August 2016). A confirmatory search in PubMed was performed in November 2016, but yielded no further relevant results.

**Type of studies**

***Inclusion criteria***

- Scientific articles published in peer-reviewed journals
- Randomized controlled trials
- Prospective observational studies
- Data availability of at least one outcome of interest

***Exclusion criteria***

- General
- Unclear or unsuitable study design
- Reported in languages different from those listed above
- Not reporting original data on outcomes of interest
- Reporting on studies conducted on non-target population (i.e., ICU-Sedation) or with not considered anaesthesia maintenance protocols (i.e., use of propofol plus inhalational anaesthesia, or any other anaesthetic combination during maintenance; change in anaesthesia procedure during procedure) or difference in the standard regimen in both groups (i.e., differences in the use of analgesics between groups)
- Exclusion from primary analyses
- Observational studies
- Quasi-randomized trials

**Types of participants**

***Inclusion criteria***

- Only human studies
- Both adults (no restriction on age) and children of age > 1 month

**Types of intervention**

***Inclusion criteria***

- General anaesthesia for surgical intervention, with anaesthesia induced with any drug or drug combination and administration route, but
- Maintained with only propofol-based intravenous anaesthetic (TIVA) or an inhalational anaesthetic (IA), used according to approved indications (on-label use)

***Exclusion criteria***

- Anaesthetic combinations/associations during maintenance
- Anaesthetic switches during the procedure
- Differences in the programmed perioperative drug regimen (excluding sedatives of interest) between treatment groups that could interfere with the valid interpretation of outcomes (e.g., α2-adrenergic agonists, antiemetics, muscle relaxants, analgesics)

**Comparators**

***Inclusion criteria***

- Inclusion criteria: Propofol vs. a single inhalational anaesthetic of interest (sevoflurane, desflurane or isoflurane)

***Exclusion criteria***

- Enflurane excluded, since the use of this agent is now uncommon and declining; it has already been withdrawn from many countries

**Settings**

***Inclusion criteria***

- Surgical intervention for in-patients or out-patients (ambulatory or day-case settings)

# Types of outcome measures

**Efficacy outcomes**

- Time to tracheal extubation, minutes recorded from anaesthetic administration stop to successful weaning from ventilator
- Time to recovery, minutes recorded from anaesthetic administration stop to recovery defined as
  - awakening (opening eyes/emergence)
  - respiratory recovery
  - orientation
  - reach a recovery score (e.g., Aldrete score ≥ 9).
  - follow simple instructions (e.g., verbal commands, ability to state name/birth date).
- Time in the post-anaesthetic care unit (PACU) or equivalent, minutes recorded from end of surgery to transfer to ICU/ward or discharge
- Incidence proportion of accidental, unintended awakenings during operation, number of patients
- Length of hospital stay (in days)
- Patient satisfaction, as reported in the paper

Data were extracted in the metric used by the authors; all times will be converted into minutes or days for quantitative pooling.

**Safety/tolerability outcomes**

- Number of hospital readmissions or conversion to in-patient in case of ambulatory surgery
- Incidence proportion (number of patients) of post-operative nausea and vomiting (PONV) and severity level (mild/moderate/severe)
- Cardiac function/haemodynamics (as described in publications)
- Incidence proportion (number of patients) of haemodynamic instability requiring unplanned intervention (e.g. vasopressor use)
- Incidence proportion (number of patients) of agitation on emergence (as described in publications)
- Neurological/cognitive recovery indicators (as described in publications)
- Incidence proportion (number of patients) of malignant hyperthermia
- Incidence proportion (number of patients) of propofol infusion syndrome
- Hospital mortality
- Incidence proportion (number of patients) and severity of post-operative pain (mild/moderate/severe)
- Incidence proportion (number of patients) and severity of post-anaesthetic shivering (mild/moderate/severe)
- Incidence proportion (number of patients) of any other grade III/IV (as defined in the Common Terminology Criteria for Adverse Events - CTCAE - Version 4.0) adverse event observed in more than 1% of participants in at least one of the comparator arms

**Drug and hospital resource consumption**

- Analgesic consumption (in the metric used by the original publications)
- Muscle relaxant consumption (in the metric used by the original publications)

**Prioritization of outcomes**

The primary outcome was PONV. Secondary key outcomes were time to recovery, post-operative pain, agitation on emergence, post-anaesthetic shivering, hospital length of stay and haemodynamic instability requiring unplanned interventions. Additional outcomes on efficacy, safety and resource consumption are listed above (Section 2).

# Study identification and selection

Literature search strategies were developed using medical subject headings (MeSH) and text words related to surgery with general anaesthesia. The search strategy was designed according to the PICOS (Patients, Intervention, Comparator, Outcome, Settings) algorithm in cooperation with a health science librarian (with expertise in systematic reviews) at the University of Torino in order to detect all relevant RCTs. There were no restrictions or filters used; the exclusion was based on the selection process defined by the eligibility criteria.

**Search strategies**

We searched MEDLINE (Pubmed interface), EMBASE (Elsevier interface), and the Cochrane Central Register of Controlled Trials (Wiley interface) in order to identify randomized trials or observational studies. Reports were included with a publication date between 1 January 1985 and the date of the last search (i.e., 1 August 2016). The search was rerun towards the end of the review in November 2016, in order to identify any relevant new material made available in the meantime. The keywords for the search were: “volatile gas”, “inhalation anaesthesia”, “sevoflurane”, “desflurane”, “isoflurane”, “propofol”, “randomized controlled trial”, “observational studies”. According to their peculiarities, each database was searched with a specific string developed on these keywords.

The following exemplifies the search strategy in MEDLINE via the Pubmed interface. As suggested by the Cochrane recommendation on Systematic Reviews, no limits were imposed on the search and exclusion/inclusion criteria were checked manually on the hits.

1. (anaesthe*[tiab] OR anaesthe*[tiab]) AND
2. (volatile[tiab] OR inhal*[tiab] OR gas[tiab] OR gases[tiab] OR maint*[tiab]) OR
3. ("Anaesthetics, Inhalation"[Mesh] OR "Anaesthetics, Inhalation"[Pharmacological Action] OR "Anaesthesia, Inhalation"[Mesh]) OR
4. (("sevoflurane"[Supplementary Concept]) OR "Isoflurane "[Mesh]) OR "desflurane"[Supplementary Concept] OR sevoflurane[tiab] OR isoflurane[tiab] OR desflurane[tiab]) AND
5. ("Propofol"[Mesh] OR propofol[tiab]) AND
6. ("randomized controlled trial"[ptyp] OR random*[tiab] OR "Observational Study"[Publication Type] OR Observational[tiab] OR "Prospective Studies"[Mesh])

To ensure literature saturation, we scanned the reference lists of studies to be included after the assessment of eligibility criteria. Furthermore, previous reviews and meta-analyses on the subject were checked in order to identify any new relevant record.

**Selection process**

In the first phase of the selection, two reviewers independently screened title and abstract of all identified publications against eligibility criteria. Subsequently, the full text of studies assessed as potentially eligible was reviewed to ascertain at the required level of detail the fulfilment of eligibility criteria in order to finalize the decision. The reviewers categorized each publication as acceptable/reconsider/excluded.

Conflicting opinions on eligibility and all cases classified as to be reconsidered were discussed with a third reviewer, after having consulted the original publication authors for clarification if necessary. The reason for the exclusion or any doubt/incertitude on the inclusion was documented at the end of each phase of the selection.

# Data extraction and quality assessment

Two reviewers independently extracted pre-defined data on study population, sample size, interventions, comparators, potential biases in the conduct of the trial, and outcomes. The reviewers tabulated the data extracted from the original publications in a predefined standardized collection grid. Any disagreement was resolved through discussion and, if necessary, in consultation with the publication authors who were contacted for further information. For outcomes shown in graphical format only, we extracted numerical values using the software Engauge® to digitize the curves. Outcomes were reported according to SI units (or prevalent in clinical practice for parameters not managed by SI); for outcomes reported in a different unit in the original paper, the conversion was made based on SI unit conversion suggestions (U.S. Department of Commerce 2006). Standard error of the mean (SEM) values were transformed into standard deviation (SD) with$SEM=SD/\sqrt{n}$, where $n$ denotes the sample size. Data reported as median and interquartile range were converted into estimated mean and SD using formulas suggested in Wan et al. 2014.

In order to facilitate the assessment of risk of bias, two reviewers independently collected information using the Cochrane Collaboration tool for assessing the risk of bias (low/high) of each included study and covered the aspects: random sequence generation, allocation concealment, blinding of participants and personnel, blinding of assessments (objective/subjective outcomes), attrition bias (short/long term) and selective reporting. If there was insufficient detail reported in the study, we judged the risk of bias as ‘unclear’ and the original study investigators were contacted for more information if deemed necessary. These judgments were made independently by two reviewers based on the Cochrane Handbook (Higgins and Green 2011).

# Statistical analysis

The narrative synthesis was provided with information presented in the text and tables to summarize and explain the characteristics and findings of included studies. If studies were sufficiently homogeneous in terms of design and comparator, data were statistically combined through meta-analytic techniques provided that outcomes were reported in at least two studies. All methods applied, including but not limited to the ones that were applied in the present project, are thoroughly detailed and discussed in the Cochrane Handbook (Higgins and Green 2011). Reporting of results was according to the PRISMA reporting guideline [Moher D PLoS Med 2009].

We computed common estimates from fixed-effect models using the inverse-variance and Mantel-Haenszel approach for continuous/count and dichotomous outcomes, respectively. In the presence of high heterogeneity, random-effects models were applied using the DerSimonian and Laird method (DerSimonian and Laird 1986). We considered P<0.05 to be statistically significant. The statistical analyses were performed using the software Review Manager (RevMan 5) and R (version 3.4.1, 2017-06-30, The R Foundation for Statistical Computing).

The difference of means with 95% confidence intervals (95% CI) was calculated for continuous outcomes; in case of different measurement scales the standardized mean difference was used. For dichotomous outcomes, the risk ratio (RR) with 95% CIs was calculated.

For dichotomous and count of events data, zero cells (i.e., no event) cause problems with the computation of estimates and variances. Thus, a zero-cell correction was to be applied for studies by adding 0.5 to both groups of the respective contingency table.

To overcome a unit-of-analysis error for a study that could contribute multiple groups, we adopted the approach recommended by the Cochrane Handbook and combined groups to create single pair-wise comparisons.

In order to determine whether reporting bias was present, we determined whether a protocol for the RCTs was published before conduct. For studies with a published protocol, we evaluated whether selective reporting of outcome was present (outcome reporting bias). The potential for reporting bias was further explored by funnel plots if ≥10 studies were available.

**Subgroup analyses**

Analysis of a-priori defined subgroups was to be performed with at least 10 included studies. For this purpose, studies were stratified according to

- Volatile comparator: Sevoflurane / desflurane / isoflurane
- Patient characteristics:
- Gender (female / male)
- Age (adults, ≥ 18 years / children, <18 years)
- Nutritional status (obese / not obese)
- Setting:
- Type of surgery (cardiac/intracranial/laparoscopic/other)
- Oncological status (oncologic vs. not oncologic)
- In-patient / out-patient
- Intervention:
- Type of anaesthesia administration protocol (TCI / not TCI)
- Induction regimen (same / different)

Table of included domains for subgroup analysis:

(Abbreviations: PACU post-anaesthesia care unit, PONV post-operative nausea and vomiting, PRIS propofol infusion syndrome, TCI target controlled infusion, Sevo sevoflurane, Des desflurane, Iso isoflurane, Onco surgery on oncological procedures)

| **Outcomes** | **Age (children/**  **adults)^^[[1]](#footnote-1)^^** | **Gender (f/m)** | **Obese/ non obese ^1^** | **TCI/**  **non-TCI** | **Sevo/**  **Des/**  **Iso** | **Cardiac surgery** | **Intra-cranial surgery** | **Laparos-copic surgery** | **Other type of surgery** | **Onco/ non-onco** | **In- patient** | **Out-patient** | **Same/**  **different induction** |
| --- | --- | --- | --- | --- | --- | --- | --- | --- | --- | --- | --- | --- | --- |
| PONV | X | X | X |  | X | X | X | X | X |  |  | X | X |
| Time to recovery |  |  | X | X | X |  | X | X | X | X | X | X | X |
| Haemodynamic instability | X | X | X | X |  | X |  |  |  | X |  |  |  |
| Length of hospital stay | X | X | X | X |  | X |  | X | X |  | X | X | X |
| Post-anaesthetic shivering |  | X |  |  | X | X | X | X | X |  | X | X | X |
| Agitation on emergence | X | X | X |  | X | X | X |  |  | X |  | X | X |
| Post-operative pain |  | X |  |  |  | X | X | X | X | X | X | X |  |
| Time to tracheal extubation |  |  | X | X | X | X | X | X | X | X | X | X | X |
| Time in PACU |  |  | X | X | X |  | X | X | X | X | X | X | X |
| Neurological/cognitive recovery |  |  |  |  | X | X | X |  |  |  | X | X |  |
| Accidental awakenings | X | X | X |  |  | X | X | X | X | X | X | X | X |
| Patient satisfaction |  |  | X |  |  |  |  | X |  |  |  | X |  |
| Hospital mortality | X |  | X |  |  | X | X | X | X | X | X |  |  |
| Cardiac outcome**^1^** | X |  | X |  |  | X |  |  | X | X |  | X |  |
| Malignant hyperthermia**^1^** |  | X | X |  |  | X | X | X | X | X | X | X |  |
| PRIS**^1^** | X |  |  |  | X | X | X | X | X | X | X | X |  |
| Hospital readmissions/conversion to in-patient in case of ambulatory surgery**^1^** | X | X | X | X |  | X | X | X | X |  | X | X |  |

# Confidence in the cumulative estimate

The quality of evidence for outcomes for which the estimated pooled effect was statistically significant was assessed using the methodology of the Grading of Recommendations Assessment, Development and Evaluation (GRADE) working group (Schünemann et al. 2013) across risk of bias, consistency, directness, precision and publication bias. For this purpose, quality of evidence was categorized as high (further research is very unlikely to change our confidence in the estimate of effect), moderate (further research is likely to have an important change on the confidence in the estimate), low (further research is very likely to have an important impact on the confidence in the estimate of the effect and is likely to change the estimate), or very low (very uncertain about the estimate of the effect). GRADEpro v. 3.6.1 was used to perform the GRADE assessment for each outcome of interest.

# Bibliography

Higgins, J P T, and S Green. 2011. “Cochrane Handbook for Systematic Reviews of Interventions Version 5.1.0 [Updated March 2011].” In *The Cochrane Collaboration*, Table 7.7.a: Formulae for combining groups.

Schünemann, Holger, J Brożek, Gordon Guyatt, and A Oxman. 2013. “GRADE Handbook for Grading Quality of Evidence and Strength of Recommendations.” The GRADE Working Group. 2013. https://doi.org/10.1136/bmj.332.7549.1089.

U.S. Department of Commerce. 2006. *The International System of Units (SI) – Conversion Factors for General Use (NIST Special Publication 1038)*. Edited by Kenneth Butcher, Linda Crown, and Elizabeth J. Gentry. Technology Administration, National Institute of Standards and Technology. https://www.nist.gov/sites/default/files/documents/pml/wmd/metric/SP1038.pdf.

Wan, Xiang, Wenqian Wang, Jiming Liu, and Tiejun Tong. 2014. “Estimating the Sample Mean and Standard Deviation from the Sample Size, Median, Range And/or Interquartile Range.” *BMC Medical Research Methodology* 14 (1). https://doi.org/10.1186/1471-2288-14-135.

1. Not performed because of insufficient number of studies [↑](#footnote-ref-1)
