# Supplementary material for: Propofol vs. inhalational agents to maintain general anaesthesia in ambulatory and in-patient surgery: a systematic review and meta-analysis
Source: BMC Anesthesiol. 2018 Nov 8;18:162. doi: 10.1186/s12871-018-0632-3 (PMC6225663; doi:10.1186/s12871-018-0632-3)
Supplement: Supplementary file 2 — This file gives additional information on the characteristics of all studies included in the primary meta-analysis. (DOCX 134 kb) [file 12871_2018_632_MOESM2_ESM.docx]

**Additional file 2**

**_________________________________________________________________________________**

**Table:** Characteristics of studies included in the primary meta-analysis

| **Study** | **Comparison** | **Type of surgery** | **Number of patients** | **Country** | **Setting** |
| --- | --- | --- | --- | --- | --- |
| Abd El-Hakeem-2003 | Propofol vs sevoflurane | Other | 30 | Egypt | Not reported |
| Acar-2015 | Propofol vs sevoflurane | Other | 40 | Turkey | In-patient |
| Akkurt BC-2009 | Propofol vs desflurane | Laparoscopic surgery | 60 | Turkey | In-patient |
| Ankichetty SP-2011 | Propofol vs isoflurane | Other | 40 | India | In-patient |
| Attar-2014 | Propofol vs isoflurane | Other | 60 | Iran | In-patient |
| Azemati S-2013 | Propofol vs isoflurane | Laparoscopic surgery | 100 | Iran | Not reported |
| Baki ED-2013 | Propofol vs desflurane | Cardiac surgery | 40 | Turkey | In-patient |
| Ballester M-2011 | Propofol vs sevoflurane | Cardiac surgery | 38 | Spain | In-patient |
| Banevičius G-2010 | Propofol vs sevoflurane | Intracranial surgery | 130 | Lithuania | In-patient |
| Bassuoni AS-2012 | Propofol vs sevoflurane | Other | 126 | Egypt | In-patient |
| Bayer-Berger MM-1989 | Propofol vs isoflurane | Intracranial surgery | 32 | Switzerland | In-patient |
| Beck DH-2001 | Propofol vs sevoflurane | Other | 38 | Germany | In-patient |
| Beck-Schimmer-2015 | Propofol vs sevoflurane | Other | 98 | Switzerland | In-patient |
| Beck-Schimmer-2016 | Propofol vs desflurane | Other | 460 | Switzerland | In-patient |
| Bein B-2005 | Propofol vs sevoflurane | Cardiac surgery | 50 | Germany | In-patient |
| Bettex DA-2014 | Propofol vs sevoflurane | Cardiac surgery | 21 | Switzerland | In-patient |
| Beule AG-2007 | Propofol vs sevoflurane | Laparoscopic surgery | 46 | Germany | In-patient |
| Biboulet P-2012 | Propofol vs sevoflurane | Other | 30 | France | In-patient |
| Bischoff P-1998 | Propofol vs isoflurane | Laparoscopic surgery | 15 | Germany | In-patient |
| Blobner M-1994 | Propofol vs isoflurane | Laparoscopic surgery | 34 | Germany | In-patient |
| Boisseau N-2002 | Propofol vs sevoflurane | Other | 24 | France | Not reported |
| Boisson-Bertrand D-1990 | Propofol vs isoflurane | Other | 43 | France | Not reported |
| Bostek CC-1992 | Propofol vs isoflurane | Other | 43 | USA | In-patient |
| Braun JP-2005 | Propofol vs desflurane | Laparoscopic surgery | 40 | Germany | In-patient |
| Braz MG-2013 | Propofol vs isoflurane | Other | 30 | Brazil | Not reported |
| Bruegger D-2002 | Propofol vs sevoflurane | Other | 20 | Germany | In-patient |
| Cai-2012 | Propofol vs isoflurane | Other | 2000 | China | In-patient |
| Carles M-2008 | Propofol vs sevoflurane | Other | 20 | France | In-patient |
| Caverni V-2005 | Propofol vs desflurane | Intracranial surgery | 80 | Italy | In-patient |
| Caverni V-2005 | Propofol vs sevoflurane | Intracranial surgery | 80 | Italy | In-patient |
| ÇELiK-2011 | Propofol vs sevoflurane | Other | 100 | Turkey | In-patient |
| Chaaban MR-2013 | Propofol vs sevoflurane | Laparoscopic surgery | 33 | USA | In-patient |
| Chan-2009 | Propofol vs desflurane | Other | 50 | China | In-patient |
| Chan-2009 | Propofol vs sevoflurane | Other | 50 | China | In-patient |
| Chen HP-2013 | Propofol vs sevoflurane | Other | 84 | Taiwan | In-patient |
| Cheng SS-2008 | Propofol vs isoflurane | Other | 40 | USA | In-patient |
| Chung F-2000 | Propofol vs isoflurane | Other | 569 | 14 countries | Not reported |
| Cinnella G-2007 | Propofol vs desflurane | Laparoscopic surgery | 40 | Italy | Not reported |
| Citerio G-2012 | Propofol vs sevoflurane | Intracranial surgery | 274 | Italy | In-patient |
| Cok OY-2011 | Propofol vs isoflurane | Intracranial surgery | 40 | Turkey | In-patient |
| Conzen PF-2003 | Propofol vs sevoflurane | Cardiac surgery | 20 | Germany | In-patient |
| Cromheecke S-2006 | Propofol vs sevoflurane | Cardiac surgery | 30 | Belgium | In-patient |
| Dabir S-2015 | Propofol vs isoflurane | Other | 88 | Iran | In-patient |
| De Conno E-2009 | Propofol vs sevoflurane | Other | 54 | Switzerland | In-patient |
| De Hert SG-2002 | Propofol vs sevoflurane | Cardiac surgery | 20 | Belgium | In-patient |
| De Hert SG-2003 | Propofol vs isoflurane | Cardiac surgery | 15 | Belgium | In-patient |
| De Hert SG-2003 | Propofol vs sevoflurane | Cardiac surgery | 30 | Belgium | In-patient |
| De Hert SG-2004 | Propofol vs desflurane | Cardiac surgery | 120 | Belgium | In-patient |
| De Hert SG-2004 | Propofol vs sevoflurane | Cardiac surgery | 120 | Belgium | In-patient |
| Di Iorio C-2010 | Propofol vs sevoflurane | Laparoscopic surgery | 30 | Italy | In-patient |
| Ebert TJ-2000 | Propofol vs desflurane | Other | 52 | USA | In-patient |
| Ebert TJ-2000 | Propofol vs sevoflurane | Other | 52 | USA | In-patient |
| Eroglu A-2003 | Propofol vs sevoflurane | Intracranial surgery | 40 | Turkey | In-patient |
| Ewaldsson CA-2005 | Propofol vs isoflurane | Other | 29 | Sweden | In-patient |
| Fabregas N-1995 | Propofol vs isoflurane | Intracranial surgery | 58 | Spain | In-patient |
| Ferber-Viart C-1998 | Propofol vs isoflurane | Other | 10 | France | Out-patient |
| Flier S-2010 | Propofol vs isoflurane | Cardiac surgery | 84 | Netherlands | In-patient |
| Fukuoka N-2009 | Propofol vs sevoflurane | Other | 32 | Japan | In-patient |
| Fung NY-2008 | Propofol vs sevoflurane | Other | 20 | Hong Kong | In-patient |
| Gazelius B-2002 | Propofol vs isoflurane | Other | 12 | Sweden | In-patient |
| Goerlich TM-2000 | Propofol vs sevoflurane | Other | 180 | Germany | In-patient |
| Gokce BM-2007 | Propofol vs desflurane | Other | 40 | Turkey | In-patient |
| Gomez-Rivera F-2012 | Propofol vs sevoflurane | Laparoscopic surgery | 23 | USA | Out-patient |
| Gouda N-2003 | Propofol vs sevoflurane | Other | 40 | Egypt | Out-patient |
| Gravel NR-1999 | Propofol vs sevoflurane | Cardiac surgery | 30 | Canada | In-patient |
| Grottke O-2004 | Propofol vs desflurane | Intracranial surgery | 36 | Germany | In-patient |
| Grundmann U-2001 | Propofol vs desflurane | Laparoscopic surgery | 50 | Germany | In-patient |
| Guarracino F-2006 | Propofol vs desflurane | Cardiac surgery | 112 | Italy | In-patient |
| Güler S-2014 | Propofol vs sevoflurane | Laparoscopic surgery | 40 | Turkey | In-patient |
| Guzzetti S-2015 | Propofol vs sevoflurane | Intracranial surgery | 53 | Italy | In-patient |
| Hammouda-2013 | Propofol vs sevoflurane | Other | 40 | Egypt | In-patient |
| Hans P-2008 | Propofol vs sevoflurane | Intracranial surgery | 34 | Belgium | In-patient |
| Hellwagner K-2003 | Propofol vs isoflurane | Other | 50 | Austria | Out-patient |
| Hernández-2015 | Propofol vs sevoflurane | Intracranial surgery | 40 | Spain | In-patient |
| Hernández-Palazón J-2006 | Propofol vs sevoflurane | Intracranial surgery | 90 | Spain | In-patient |
| Höcker J-2006 | Propofol vs sevoflurane | Other | 103 | Germany | Out-patient |
| Hofer CK-2003 | Propofol vs sevoflurane | Other | 301 | Switzerland | In-patient |
| Holzer A-2003 | Propofol vs sevoflurane | Intracranial surgery | 30 | Austria | In-patient |
| Horng HC-2008 | Propofol vs desflurane | Laparoscopic surgery | 60 | Taiwan | In-patient |
| Horng HC-2008 | Propofol vs sevoflurane | Laparoscopic surgery | 60 | Taiwan | In-patient |
| Huang CH-2008 | Propofol vs isoflurane | Other | 30 | Taiwan | In-patient |
| Huang Z-2011 | Propofol vs isoflurane | Cardiac surgery | 60 | China | In-patient |
| Hüpfl M-2008 | Propofol vs sevoflurane | Other | 30 | Australia | In-patient |
| Husedzinovic I-2003 | Propofol vs sevoflurane | Laparoscopic surgery | 40 | Croatia | Out-patient |
| Huseidzinović I-2007 | Propofol vs sevoflurane | Cardiac surgery | 32 | Croatia | In-patient |
| Hwang JW-2013 | Propofol vs desflurane | Laparoscopic surgery | 50 | Korea | Out-patient |
| Hwang JW-2013 | Propofol vs desflurane | Laparoscopic surgery | 50 | Korea | Out-patient |
| Ingelmo PM-2007 | Propofol vs sevoflurane | Other | 113 | Italy | In-patient |
| Ionescu-2013 | Propofol vs isoflurane | Laparoscopic surgery | 88 | Romania | Not reported |
| Ishii-2016 | Propofol vs sevoflurane | Other | 59 | Japan | In-patient |
| Ivani G-1996 | Propofol vs isoflurane | Other | 24 | Italy | Not reported |
| Iwata M-2008 | Propofol vs sevoflurane | Other | 52 | Japan | In-patient |
| Jellish WS-1995 | Propofol vs isoflurane | Other | 68 | USA | Out-patient |
| Jellish WS-1999 | Propofol vs isoflurane | Other | 79 | USA | In-patient |
| Jeong CW-2012 | Propofol vs sevoflurane | Laparoscopic surgery | 24 | Korea | In-patient |
| Jeong CW-2012 | Propofol vs sevoflurane | Laparoscopic surgery | 24 | Korea | In-patient |
| Ji FH-2011 | Propofol vs sevoflurane | Laparoscopic surgery | 28 | China | In-patient |
| Jia L-2015 | Propofol vs sevoflurane | Cardiac surgery | 67 | China | In-patient |
| Jiang A-2016 | Propofol vs sevoflurane | Laparoscopic surgery | 100 | China | Out-patient |
| Jokela RM-2000 | Propofol vs sevoflurane | Other | 120 | Finland | In-patient |
| Kalimeris K-2013 | Propofol vs sevoflurane | Other | 44 | Greece | In-patient |
| Kawamura T-2006 | Propofol vs sevoflurane | Cardiac surgery | 24 | Japan | In-patient |
| Kawano H-2016 | Propofol vs sevoflurane | Laparoscopic surgery | 84 | Japan | Out-patient |
| Keller C-2005 | Propofol vs isoflurane | Other | 40 | Australia | In-patient |
| Keller C-2005 | Propofol vs sevoflurane | Other | 40 | Australia | In-patient |
| Khanjani-2014 | Propofol vs isoflurane | Other | 90 | Iran | In-patient |
| Kim H-2012 | Propofol vs sevoflurane | Other | 70 | Korea | In-patient |
| Kim JY-2014 | Propofol vs desflurane | Other | 40 | Korea | Not reported |
| Kim TY-2011 | Propofol vs sevoflurane | Cardiac surgery | 94 | Korea | In-patient |
| Kim YS-2015 | Propofol vs desflurane | Other | 100 | Korea | In-patient |
| Kirov MY-2007 | Propofol vs isoflurane | Cardiac surgery | 24 | Russia | In-patient |
| Kleinsasser A-2000 | Propofol vs sevoflurane | Other | 30 | Austria | Out-patient |
| Kochs E-2000 | Propofol vs isoflurane | Other | 553 | 14 countries | Out-patient |
| Kostopanagiotou G-2011 | Propofol vs sevoflurane | Other | 32 | Greece | In-patient |
| Kotani N-1998 | Propofol vs isoflurane | Other | 60 | Japan | In-patient |
| Kotani N-1999 | Propofol vs isoflurane | Other | 40 | Japan | In-patient |
| Krueper-1997 | Propofol vs isoflurane | Laparoscopic surgery | 60 | Germany | Not reported |
| Kubota-1999 | Propofol vs isoflurane | Cardiac surgery | 24 | Japan | In-patient |
| Kumar-2016 | Propofol vs sevoflurane | Intracranial surgery | 60 | India | In-patient |
| Kwak HJ-2011 | Propofol vs sevoflurane | Laparoscopic surgery | 50 | Korea | In-patient |
| Landoni G-2014 | Propofol vs sevoflurane | Cardiac surgery | 200 | Italy | In-patient |
| Launo-1994 | Propofol vs isoflurane | Other | 100 | Italy | In-patient |
| Lauta E-2010 | Propofol vs sevoflurane | Intracranial surgery | 302 | Italy | In-patient |
| Law-Koune JD-2006 | Propofol vs sevoflurane | Cardiac surgery | 18 | France | In-patient |
| Ledderose H-1988 | Propofol vs isoflurane | Other | 50 | Germany | In-patient |
| Ledowski T-2005 | Propofol vs sevoflurane | Other | 43 | Germany | Not reported |
| Lee DW-2011 | Propofol vs sevoflurane | Other | 62 | Korea | In-patient |
| Lee JH-2014 | Propofol vs desflurane | Other | 52 | Korea | In-patient |
| Lee JH-2014 | Propofol vs sevoflurane | Other | 49 | Korea | In-patient |
| Lee SH-2015 | Propofol vs sevoflurane | Other | 416 | Korea | In-patient |
| Lee WK-2015 | Propofol vs desflurane | Other | 76 | Korea | In-patient |
| Lehavi-2015 | Propofol vs sevoflurane | Laparoscopic surgery | 30 | Israel | In-patient |
| Leslie K-2009 | Propofol vs desflurane | Other | 300 | Australia | Out-patient |
| Li M-2012 | Propofol vs sevoflurane | Laparoscopic surgery | 60 | China | Out-patient |
| Lindqvist M-2014 | Propofol vs desflurane | Other | 59 | Sweden | Out-patient |
| Liu EH-2005 | Propofol vs isoflurane | Intracranial surgery | 60 | Singapore | In-patient |
| Liu TC-2014 | Propofol vs isoflurane | Other | 62 | China | In-patient |
| Liu X-2016 | Propofol vs sevoflurane | Cardiac surgery | 36 | China | In-patient |
| Liu Y-2013 | Propofol vs sevoflurane | Other | 107 | China | In-patient |
| Longás Valién J-2004 | Propofol vs sevoflurane | Other | 40 | Spain | In-patient |
| Longás Valién J-2004 | Propofol vs sevoflurane | Other | 40 | Spain | In-patient |
| Loop T-2002 | Propofol vs desflurane | Other | 90 | Germany | In-patient |
| Loop T-2002 | Propofol vs sevoflurane | Other | 90 | Germany | In-patient |
| Lopez Alvarez S-2001 | Propofol vs desflurane | Other | 70 | Spain | Out-patient |
| Lorsomradee S-2006 | Propofol vs sevoflurane | Cardiac surgery | 320 | Belgium | In-patient |
| Luginbuhl M-2003 | Propofol vs desflurane | Other | 160 | Switzerland | In-patient |
| Luntz SP-2004 | Propofol vs sevoflurane | Other | 96 | Germany | In-patient |
| Lurati Buse GA-2012 | Propofol vs sevoflurane | Other | 385 | Switzerland | In-patient |
| Mahdavi-2015 | Propofol vs sevoflurane | Cardiac surgery | 84 | Iran | In-patient |
| Mahli-2011 | Propofol vs desflurane | Other | 40 | Iran | In-patient |
| Mahmoud K-2011 | Propofol vs isoflurane | Other | 50 | Turkey | Out-patient |
| Mandell MS-2003 | Propofol vs desflurane | Other | 21 | Egypt | In-patient |
| Margarit SC-2014 | Propofol vs isoflurane | Other | 60 | USA | In-patient |
| Markovic Bozic-2016 | Propofol vs sevoflurane | Intracranial surgery | 40 | Romania | In-patient |
| Martikainen-1998 | Propofol vs desflurane | Other | 80 | Slovenia | In-patient |
| Martikainen-1998 | Propofol vs isoflurane | Other | 70 | Finland | Out-patient |
| Martikainen-2000 | Propofol vs desflurane | Other | 80 | Finland | Out-patient |
| Martikainen-2000 | Propofol vs isoflurane | Other | 70 | Finland | Out-patient |
| Martikainen M-2000 | Propofol vs desflurane | Other | 118 | Finland | Out-patient |
| Martikainen M-2000 | Propofol vs isoflurane | Other | 118 | Finland | Out-patient |
| Martin-Castro C-2008 | Propofol vs sevoflurane | Other | 120 | Finland | Out-patient |
| Mazoti MA-2013 | Propofol vs isoflurane | Other | 34 | Spain | In-patient |
| Mei W-2014 | Propofol vs sevoflurane | Laparoscopic surgery | 148 | Brazil | In-patient |
| Mencke T-2013 | Propofol vs sevoflurane | Other | 58 | China | In-patient |
| Monedero-1994 | Propofol vs isoflurane | Other | 42 | Germany | In-patient |
| Montazeri-2015 | Propofol vs isoflurane | Other | 63 | Spain | Not reported |
| Montazeri-2015 | Propofol vs isoflurane | Other | 71 | Iran | In-patient |
| Mousa-2013 | Propofol vs sevoflurane | Other | 40 | Iran | In-patient |
| Mroziński P-2014 | Propofol vs sevoflurane | Cardiac surgery | 60 | Egypt | In-patient |
| Muralidhar-2008 | Propofol vs isoflurane | Cardiac surgery | 20 | Poland | In-patient |
| Muralidhar-2008 | Propofol vs isoflurane | Cardiac surgery | 20 | India | In-patient |
| Musialowicz T-2007 | Propofol vs isoflurane | Cardiac surgery | 24 | India | In-patient |
| Mutch WA-1995 | Propofol vs isoflurane | Cardiac surgery | 27 | Finland | In-patient |
| Nakada T-2010 | Propofol vs isoflurane | Other | 60 | Japan | In-patient |
| Nakada T-2010 | Propofol vs sevoflurane | Other | 60 | Canada | In-patient |
| Nakada T-2010 | Propofol vs sevoflurane | Other | 60 | Japan | In-patient |
| Naoki Kotani-1999 | Propofol vs isoflurane | Other | 40 | Japan | In-patient |
| Oberer C-2005 | Propofol vs sevoflurane | Other | 70 | Japan | Not reported |
| Oddby-Muhrbeck E-1994 | Propofol vs isoflurane | Other | 60 | Austria | Not reported |
| Oddby-Muhrbeck E-1994 | Propofol vs isoflurane | Other | 60 | Sweden | In-patient |
| Ogurlu M-2014 | Propofol vs sevoflurane | Other | 80 | Sweden | In-patient |
| Ohtani N-2008 | Propofol vs sevoflurane | Other | 30 | Turkey | In-patient |
| Oikkonen M-1994 | Propofol vs isoflurane | Laparoscopic surgery | 30 | Japan | In-patient |
| Ortiz J-2014 | Propofol vs desflurane | Laparoscopic surgery | 38 | USA | In-patient |
| Ortiz J-2014 | Propofol vs isoflurane | Laparoscopic surgery | 36 | USA | In-patient |
| Ortiz J-2014 | Propofol vs sevoflurane | Laparoscopic surgery | 36 | Finland | Not reported |
| Ozcan PE-2007 | Propofol vs isoflurane | Other | 50 | USA | In-patient |
| Ozdemır-2013 | Propofol vs sevoflurane | Laparoscopic surgery | 42 | Turkey | In-patient |
| Oztürk-2013 | Propofol vs desflurane | Other | 40 | Turkey | Not reported |
| Pagnin-1992 | Propofol vs isoflurane | Cardiac surgery | 20 | Turkey | In-patient |
| Panditrao MM-2013 | Propofol vs sevoflurane | Other | 40 | Italy | In-patient |
| Parker FC-2004 | Propofol vs isoflurane | Cardiac surgery | 236 | Australia | In-patient |
| Parker FC-2004 | Propofol vs sevoflurane | Cardiac surgery | 236 | India | Out-patient |
| Paventi S-2001 | Propofol vs sevoflurane | Other | 180 | Australia | In-patient |
| Petersen KD-2002 | Propofol vs isoflurane | Intracranial surgery | 117 | Denmark | In-patient |
| Petersen KD-2002 | Propofol vs sevoflurane | Intracranial surgery | 117 | Italy | Not reported |
| Petersen KD-2003 | Propofol vs isoflurane | Intracranial surgery | 117 | Denmark | In-patient |
| Petersen KD-2003 | Propofol vs sevoflurane | Intracranial surgery | 117 | Denmark | In-patient |
| Phillips AS-1994 | Propofol vs isoflurane | Cardiac surgery | 64 | Denmark | In-patient |
| Pieters BJ-2010 | Propofol vs sevoflurane | Other | 38 | Northen Ireland | In-patient |
| Pokkinen SM-2014 | Propofol vs sevoflurane | Laparoscopic surgery | 148 | USA | Out-patient |
| Potocnik I-2015 | Propofol vs sevoflurane | Other | 36 | Finland | In-patient |
| Prasanna-2010 | Propofol vs isoflurane | Other | 60 | Slovenia | In-patient |
| Procaccini B-1996 | Propofol vs isoflurane | Cardiac surgery | 30 | Oman | Out-patient |
| Qiao Y-2015 | Propofol vs sevoflurane | Other | 60 | Italy | In-patient |
| Reyes-Partida-2007 | Propofol vs desflurane | Other | 76 | China | In-patient |
| Rohan D-2005 | Propofol vs sevoflurane | Other | 30 | Mexico | In-patient |
| Rowbotham DJ-1998 | Propofol vs isoflurane | Other | 233 | USA | Out-patient |
| Salihoglu Z-2001 | Propofol vs sevoflurane | Other | 40 | UK | In-patient |
| Salihoğlu Z-2004 | Propofol vs sevoflurane | Other | 40 | Turkey | In-patient |
| Schafer R-2002 | Propofol vs sevoflurane | Other | 40 | Turkey | In-patient |
| Schilling T-2011 | Propofol vs desflurane | Other | 42 | Germany | In-patient |
| Schilling T-2011 | Propofol vs sevoflurane | Other | 42 | Germany | Out-patient |
| Schmidt J-2001 | Propofol vs sevoflurane | Other | 120 | Germany | In-patient |
| Schneider G-2003 | Propofol vs isoflurane | Other | 80 | Germany | In-patient |
| Schricker T-2001 | Propofol vs desflurane | Other | 12 | USA | In-patient |
| Schwarzkopf K-2009 | Propofol vs sevoflurane | Other | 54 | Canada | In-patient |
| Sharifian Attar A-2014 | Propofol vs isoflurane | Other | 60 | Germany | In-patient |
| Shen L-2014 | Propofol vs desflurane | Laparoscopic surgery | 180^[[1]](#footnote-1)^ | Iran | In-patient |
| Shen L-2014 | Propofol vs sevoflurane | Laparoscopic surgery |  | Iran | In-patient |
| Shin SW-2010 | Propofol vs sevoflurane | Other | 98 | China | Out-patient |
| Shin SW-2010 | Propofol vs sevoflurane | Other | 88 | Korea | In-patient |
| Shinn-2011 | Propofol vs sevoflurane | Laparoscopic surgery | 35 | Korea | In-patient |
| Sirvinskas-2015 | Propofol vs sevoflurane | Cardiac surgery | 72 | Korea | In-patient |
| Sneyd JR-2005 | Propofol vs sevoflurane | Intracranial surgery | 50 | Lithuania | In-patient |
| Song JC-2010 | Propofol vs sevoflurane | Other | 100 | UK | In-patient |
| Song JC-2013 | Propofol vs sevoflurane | Other | 102 | China | In-patient |
| Sorbara C-1995 | Propofol vs isoflurane | Cardiac surgery | 30 | China | In-patient |
| Speicher A-1995 | Propofol vs isoflurane | Other | 63 | Italy | In-patient |
| Stevanovic PD-2008 | Propofol vs sevoflurane | Laparoscopic surgery | 60 | Germany | In-patient |
| Struys MM-2002 | Propofol vs sevoflurane | Laparoscopic surgery | 40 | Serbia | Out-patient |
| Sudheer PS-2006 | Propofol vs isoflurane | Intracranial surgery | 40 | Belgium | In-patient |
| Sugata A-2012 | Propofol vs sevoflurane | Intracranial surgery | 24 | UK | In-patient |
| Suryaprakash S-2013 | Propofol vs desflurane | Cardiac surgery | 88 | India | In-patient |
| Suryaprakash S-2013 | Propofol vs sevoflurane | Cardiac surgery | 84 | Japan | In-patient |
| Tan T-2010 | Propofol vs sevoflurane | Laparoscopic surgery | 80 | India | In-patient |
| Tang N-2014 | Propofol vs sevoflurane | Other | 200 | Ireland | Out-patient |
| Tramèr MR-1998 | Propofol vs isoflurane | Other | 77 | China | In-patient |
| Tsuchiya M-2008 | Propofol vs sevoflurane | Laparoscopic surgery | 36 | Switzerland | In-patient |
| Tsuchiya M-2008 | Propofol vs sevoflurane | Other | 37 | Japan | In-patient |
| Valencia L-2014 | Propofol vs sevoflurane | Other | 48 | Japan | In-patient |
| Van der Linden PJ-2010 | Propofol vs sevoflurane | Cardiac surgery | 40 | Spain | In-patient |
| Vernon JM-1995 | Propofol vs isoflurane | Other | 50 | Belgium | In-patient |
| Von Dossow V-2007 | Propofol vs isoflurane | Other | 28 | USA | Not reported |
| Webster NR-1994 | Propofol vs isoflurane | Other | 26 | Germany | In-patient |
| Weninger-2004 | Propofol vs sevoflurane | Intracranial surgery | 34 | Germany | In-patient |
| Wilhelm W-1998 | Propofol vs desflurane | Laparoscopic surgery | 40 | UK | In-patient |
| Xia Z-2006 | Propofol vs isoflurane | Cardiac surgery | 36 | Germany | In-patient |
| Xia Z-2006 | Propofol vs isoflurane | Cardiac surgery | 36 | China | In-patient |
| Xu WY-2014 | Propofol vs sevoflurane | Other | 40 | China | In-patient |
| Yang-2010 | Propofol vs isoflurane | Other | 60 | China | In-patient |
| Yang WC-2015 | Propofol vs sevoflurane | Intracranial surgery | 30 | Hong Kong | In-patient |
| Yildirim V-2009 | Propofol vs isoflurane | Cardiac surgery | 40 | Turkey | In-patient |
| Yildirim V-2009 | Propofol vs sevoflurane | Cardiac surgery | 40 | China | In-patient |
| Yin J-2014 | Propofol vs sevoflurane | Other | 60 | Turkey | In-patient |
| Yoo YC-2012 | Propofol vs desflurane | Laparoscopic surgery | 62 | China | In-patient |
| Yoo YC-2014 | Propofol vs sevoflurane | Laparoscopic surgery | 66 | Korea | In-patient |
| Yoshitani K-2004 | Propofol vs isoflurane | Other | 42 | Korea | In-patient |
| Zangrillo A-2011 | Propofol vs sevoflurane | Other | 88 | Japan | Not reported |
| Zhang D-2013 | Propofol vs sevoflurane | Laparoscopic surgery | 80 | Italy | In-patient |
| Zhang L-2016 | Propofol vs sevoflurane | Other | 40 | China | In-patient |
| Zhang L-2016 | Propofol vs sevoflurane | Other | 40 | China | In-patient |

Characteristics of observational studies included (in addition to RCTs) in the secondary meta-analysis

| **Study** | **Comparison** | **Type of surgery** | **Number of patients** | **Country** | **Setting** |
| --- | --- | --- | --- | --- | --- |
| Elliott RA-2004 | Propofol vs isoflurane | Other | 383 | UK | Out-patient |
| Elliott RA-2004 | Propofol vs sevoflurane | Other | 680 | UK | Out-patient |
| Fombeur PO-2002 | Propofol vs desflurane | Other | 100 | France | In-patient |
| Lepousé C-2006 | Propofol vs desflurane | Other | 170 | France | In-patient |
| Lepousé C-2006 | Propofol vs isoflurane | Other | 454 | France | In-patient |
| Lepousé C-2006 | Propofol vs sevoflurane | Other | 684 | France | In-patient |
| Saringcarinkul A-2008 | Propofol vs isoflurane | Other | 112 | Thailand | In-patient |
| Saringcarinkul A-2008 | Propofol vs sevoflurane | Other | 172 | Thailand | In-patient |
| Slankamenac K-2012 | Propofol vs sevoflurane | Other | 227 | USA | In-patient |
| Wu ZF-2014 | Propofol vs desflurane | Other | 1405 | China | In-patient |

Figure: Number of randomized controlled trials (RCTs) included in primary meta-analysis by A) year of publication B) inhalational agent C) type of surgery D) in/out-patient setting. The total number of included RCTs was 229, whereof 31 reported multiple subgroups.


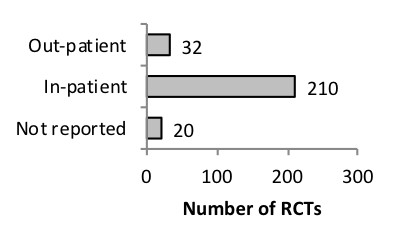

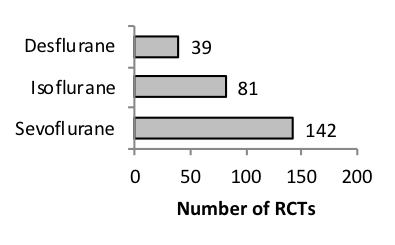

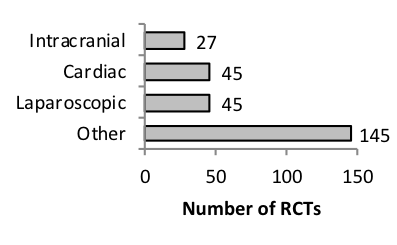


A

C

B

D


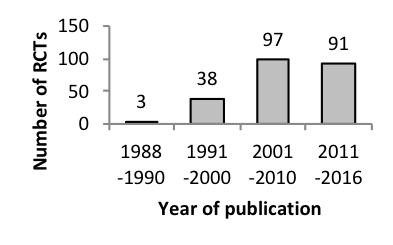


1. Group size of n=60 for propofol (common control group), sevoflurane and desflurane [↑](#footnote-ref-1)
