# Supplementary material for: Propofol vs. inhalational agents to maintain general anaesthesia in ambulatory and in-patient surgery: a systematic review and meta-analysis
Source: BMC Anesthesiol. 2018 Nov 8;18:162. doi: 10.1186/s12871-018-0632-3 (PMC6225663; doi:10.1186/s12871-018-0632-3)
Supplement: Supplementary file 3 — This file provides a summary of effect estimates for primary and secondary outcomes including subgroups of all RCTs. (DOCX 36 kb) [file 12871_2018_632_MOESM3_ESM.docx]

Additional file 3

Subgroup analysis of randomized controlled trials:

Summary of effect estimates for primary and secondary outcomes including subgroups

(Abbreviations: PACU post-anaesthesia care unit, PONV post-operative nausea and vomiting, TCI target controlled infusion, LOS length of stay, CI confidence interval, RR relative risk, MD mean difference, Std. MD standardized mean difference, M-H Mantel-Haenzsel, FE fixed effects, IV inverse variance, RE random effects, P in favour of propofol, I in favour of inhalational agents, IA inhalational agent, NS not significant)

| **Outcome/**  **subgroup** | **No. of studies** | **No. of patients** | **Effect measure (statistical model)** | **Effect estimate**  **(95% CI)** | **Effect** |
| --- | --- | --- | --- | --- | --- |
| **Haemodynamic instability** | 14 | 1327 | RR (M-H, FE) | 1.01 [0.86, 1.19] | NS |
| **Accidental awakenings** | 3 | 350 | RR (M-H, FE) | 1.36 [0.64, 2.89] | NS |
| **Muscle relaxant consumption** | 11 | 821 | Std. MD (IV, FE) | 0.18 [0.04, 0.32] | I |
| **Agitation on emergence** | 6 | 923 | RR (M-H, FE) | 0.91 [0.61, 1.34] | NS |
| **Time to awakening (minutes)** | 40 | 3064 | MD (IV, RE) | -0.01 [-0.71, 0.68] | NS |
| Sevoflurane | 23 | 1553 | MD (IV, RE) | 0.15 [-0.76, 1.06] | NS |
| Desflurane | 15 | 1078 | MD (IV, RE) | 0.65 [-0.75, 2.05] | NS |
| Other type of surgery | 21 | 1570 | MD (IV, RE) | -0.26 [-1.31, 0.79] | NS |
| In-patient | 29 | 1959 | MD (IV, RE) | 0.23 [-0.64, 1.10] | NS |
| TCI | 13 | 1186 | MD (IV, RE) | -0.16 [-1.44, 1.13] | NS |
| Non-TCI | 14 | 772 | MD (IV, RE) | -0.19 [-1.17, 0.79] | NS |
| Onco. | 12 | 1040 | MD (IV, RE) | 0.02 [-1.25, 1.28] | NS |
| Non-onco. | 12 | 743 | MD (IV, RE) | -0.26 [-1.14, 0.62] | NS |
| Same induction (P or IA) | 24 | 1941 | MD (IV, RE) | -0.33 [-1.19, 0.54] | NS |
| **Time to respiratory recovery (minutes)** | 20 | 2622 | MD (IV, RE) | 0.82 [0.20, 1.45] | I |
| Sevoflurane | 10 | 635 | MD (IV, RE) | 0.32 [-0.26, 0.89] | NS |
| In-patient | 15 | 1679 | MD (IV, RE) | 0.84 [-0.11, 1.79] | NS |
| TCI ^[[1]](#footnote-1)^ | 7 | 586 | MD (IV, RE) | 0.39 [-0.38, 1.15] | NS |
| Non-TCI | 10 | 1873 | MD (IV, RE) | 0.92 [0.35, 1.50] | I |
| Same induction (P or IA) | 14 | 2191 | MD (IV, RE) | 1.02 [0.24, 1.80] | I |
| Different induction (same as maintenance agent) ^1^ | 3 | 270 | MD (IV, RE) | 0.00 [-0.42, 0.43] | NS |
| **Time to orientation (minutes)** | 20 | 1738 | MD (IV, RE) | 1.35 [-0.12, 2.82] | NS |
| Sevoflurane | 12 | 689 | MD (IV, RE) | 0.94 [-0.45, 2.32] | NS |
| In-patient | 13 | 870 | MD (IV, RE) | 0.80 [-1.06, 2.66] | NS |
| Same induction (P or IA) | 13 | 1304 | MD (IV, RE) | 1.91 [0.20, 3.63] | I |
| Different induction (same as maintenance agent) ^1^ | 5 | 350 | MD (IV, RE) | 1.61 [-1.32, 4.53] | NS |
| **Time to reach a score (minutes)** | 12 | 2114 | MD (IV, RE) | 0.13 [-1.09, 1.35] | NS |
| **Time to follow simple instructions (minutes)** | 23 | 2497 | MD (IV, RE) | 0.46 [-0.39, 1.30] | NS |
| In-patient | 19 | 1716 | MD (IV, RE) | 0.48 [-0.61, 1.58] | NS |
| Same induction (P or IA) | 16 | 2165 | MD (IV, RE) | 0.90 [0.02, 1.78] | I |
| **Time to tracheal extubation (minutes)** | 61 | 5773 | MD (IV, RE) | 0.70 [0.03, 1.38] | I |
| Sevoflurane | 33 | 2173 | MD (IV, RE) | -0.26 [-1.30, 0.78] | NS |
| Desflurane | 18 | 1002 | MD (IV, RE) | 1.02 [-0.45, 2.50] | NS |
| Isoflurane | 27 | 2598 | MD (IV, RE) | 0.72 [-0.36, 1.81] | NS |
| Onco. | 15 | 1353 | MD (IV, RE) | 0.94 [0.12, 1.77] | I |
| Non-onco. | 32 | 2917 | MD (IV, RE) | 1.08 [-0.11, 2.28] | NS |
| TCI | 20 | 1560 | MD (IV, RE) | 0.48 [-1.06, 2.02] | NS |
| Non-TCI | 30 | 2826 | MD (IV, RE) | 0.62 [-0.21, 1.45] | NS |
| Laparoscopic | 11 | 559 | MD (IV, RE) | 0.76 [-0.40, 1.92] | NS |
| Intracranial | 8 | 763 | MD (IV, RE) | 1.96 [0.13, 3.80] | I |
| Other | 35 | 3765 | MD (IV, RE) | 0.44 [-0.44, 1.31] | NS |
| In-patient | 48 | 4017 | MD (IV, RE) | 0.86 [-0.09, 1.82] | NS |
| Out-patient | 13 | 1756 | MD (IV, RE) | 0.32 [-0.42, 1.06] | NS |
| Same induction (Neither P nor IAA) | 11 | 1249 | MD (IV, RE) | -0.96 [-3.21, 1.29] | NS |
| Same induction (P or IA) | 35 | 3610 | MD (IV, RE) | 0.90 [0.17, 1.64] | I |
| **Post-anaesthetic shivering** | 23 | 3382 | RR (M-H, FE) | 1.06 [0.92, 1.22] | NS |
| Sevoflurane | 15 | 1680 | RR (M-H, FE) | 1.15 [0.96, 1.38] | NS |
| In-patient | 16 | 1955 | RR (M-H, FE) | 1.14 [0.97, 1.34] | NS |
| Same induction (P or IA) | 15 | 2141 | RR (M-H, FE) | 1.02 [0.85, 1.23] | NS |
| **PONV** | 65 | 7043 | RR (M-H, RE) | 0.61 [0.53, 0.69] | P |
| Sevoflurane | 40 | 3892 | RR (M-H, FE) | 0.55 [0.49, 0.61] | P |
| Desflurane | 17 | 1002 | RR (M-H, FE) | 0.53 [0.44, 0.64] | P |
| Isoflurane | 15 | 2149 | RR (M-H, FE) | 0.81 [0.72, 0.92] | P |
| Female | 22 | 1688 | RR (M-H, FE) | 0.56 [0.49, 0.65] | P |
| Mixed | 39 | 5199 | RR (M-H, RE) | 0.62 [0.53, 0.73] | P |
| Laparoscopic | 21 | 1459 | RR (M-H, FE) | 0.62 [0.53, 0.72] | P |
| Other type of surgery | 36 | 4617 | RR (M-H, RE) | 0.60 [0.51, 0.71] | P |
| Out-patient | 14 | 1555 | RR (M-H, FE) | 0.82 [0.71, 0.94] | P |
| Same induction (P or IA) | 45 | 4853 | RR (M-H, RE) | 0.58 [0.50, 0.68] | P |
| PONV as primary outcome | 21 | 1720 | RR (M-H, RE) | 0.48 [0.36, 0.64] | P |
| PONV as secondary outcome | 44 | 5323 | RR (M-H, RE) | 0.66 [0.57, 0.75] | P |
| Nausea | 28 | 3254 | RR (M-H, FE) | 0.64 [0.55, 0.75] | P |
| Vomiting | 38 | 3860 | RR (M-H, FE) | 0.64 [0.54, 0.75] | P |
| **Pain score – After tracheal extubation** | 27 | 2631 | MD (IV, RE) | -0.51 [-0.81, -0.20] | P |
| Female | 10 | 1211 | MD (IV, RE) | -0.73 [-1.19, -0.27] | NS |
| Mixed | 14 | 1210 | MD (IV, RE) | -0.28 [-0.79, 0.22] | NS |
| Laparoscopic | 11 | 775 | MD (IV, RE) | -0.90 [-1.43, -0.37] | P |
| Other type of surgery | 16 | 1808 | MD (IV, RE) | -0.34 [-0.69, 0.02] | NS |
| In-patient | 21 | 2116 | MD (IV, RE) | -0.50 [-0.83, -0.18] | P |
| **Pain score - 12 to 24 hours after tracheal extubation** | 18 | 1836 | MD (IV, RE) | -0.14 [-0.31, 0.03] | NS |
| In-patient | 13 | 1522 | MD (IV, RE) | -0.11 [-0.33, 0.10] | NS |
| **Analgesic consumption** | 40 | 4192 | Std. MD (IV, RE) | 0.20 [0.03, 0.37] | I |
| **PACU time (minutes)** | 21 | 2653 | MD (IV, RE) | -2.91 [-5.47, -0.35] | P |
| Sevoflurane | 12 | 1009 | MD (IV, RE) | -4.99 [-8.81, -1.17] | P |
| TCI ^[[2]](#footnote-2)^ | 8 | 920 | MD (IV, RE) | -2.51 [-6.70, 1.68] | NS |
| Non-TCI | 12 | 1649 | MD (IV, RE) | -2.64 [-7.30, 2.02] | NS |
| Non-onco. | 10 | 1026 | MD (IV, RE) | -3.21 [-6.59, 0.18] | NS |
| Out-patient ^1^ | 9 | 1381 | MD (IV, RE) | -4.75 [-11.43, 1.94] | NS |
| In-patient | 12 | 1272 | MD (IV, RE) | 0.03 [-1.39, 1.44] | NS |
| Same induction (P or IA) | 15 | 1951 | MD (IV, RE) | -1.78 [-4.61, 1.05] | NS |
| **Cognitive assessment scales** | 7 | 387 | Std. MD (IV, RE) | 0.45 [-0.15, 1.05] | NS |
| **Post-operative cognitive dysfunction** | 4 | 674 | RR (M-H, FE) | 1.14 [0.86, 1.51] | NS |
| **Patients satisfied** | 10 | 924 | RR (M-H, FE) | 1.06 [1.01, 1.10] | P |
| **Satisfaction score** | 5 | 967 | Std. MD (IV, FE) | 0.13 [0.00, 0.26] | P |
| **Grade 3-4 adverse event** | 14 | 2046 | RR (M-H, FE) | 1.04 [0.76, 1.42] | NS |
| **Hospital mortality** | 11 | 1427 | RR (M-H, FE) | 0.78 [0.40, 1.53] | NS |
| **Hospital LOS (days)** | 30 | 2837 | MD (IV, RE) | 0.18 [-0.16, 0.53] | NS |
| TCI | 12 | 1561 | MD (IV, RE) | 0.66 [-0.47, 1.78] | NS |
| Non-TCI | 11 | 1250 | MD (IV, RE) | -0.04 [-0.11, 0.02] | NS |
| Cardiac | 14 | 1312 | MD (IV, RE) | 0.68 [-0.05, 1.40] | NS |
| Other type of surgery | 15 | 1933 | MD (IV, RE) | -0.17 [-0.36, 0.02] | NS |
| In-patient | 29 | 2738 | MD (IV, RE) | 0.16 [-0.20, 0.52] | NS |
| Same induction (Neither P nor IAA) | 10 | 740 | MD (IV, RE) | 0.25 [-0.13, 0.63] | NS |

1. Post-hoc analysis [↑](#footnote-ref-1)
2. Post-hoc analysis [↑](#footnote-ref-2)
